# Enteral Feeding During Therapeutic Hypothermia for Hypoxic–Ischemic Encephalopathy in Neonates: A Multicentre Study from Italy and Belgium

**DOI:** 10.3390/nu18193240

**Published:** 2026-10-01

**Authors:** Domenico Umberto De Rose, Iliana Bersani, Guglielmo Salvatori, Francesca Campi, Ludovica Martini, Chiara Maddaloni, Sara Ronci, Alessandra Santisi, Immacolata Savarese, Cinzia Auriti, Elena Camacho Santamaria, Anaïs De Groote, Katherine Carkeek, Giulia Iacona, Maria Roberta Cilio, Maria Paola Ronchetti, Annabella Braguglia, Olivier Danhaive, Andrea Dotta, Fiammetta Piersigilli

**Affiliations:** 1 Neonatal Intensive Care Unit, “Bambino Gesù” Children’s Hospital IRCCS, 00165 Rome, Italy; iliana.bersani@opbg.net (I.B.); guglielmo.salvatori@opbg.net (G.S.); francesca.campi@opbg.net (F.C.); chiara.maddaloni@opbg.net (C.M.); sara.ronci@opbg.net (S.R.); alessandra.santisi@opbg.net (A.S.); immacolata.savarese@opbg.net (I.S.); mariapaola.ronchetti@opbg.net (M.P.R.); andrea.dotta@opbg.net (A.D.); 2 Faculty of Medicine and Surgery, Unicamillus, Saint Camillus International University of Health and Medical Sciences, 00131 Rome, Italy; cinzia.auriti@gmail.com; 3 Division of Neonatology, Cliniques Universitaires Saint Luc, Université Catholique de Louvain, 1200 Brussels, Belgiumanais.degroote@saintluc.uclouvain.be (A.D.G.); katherine.carkeek@saintluc.uclouvain.be (K.C.); olivier.danhaive@saintluc.uclouvain.be (O.D.); fiammetta.piersigilli@saintluc.uclouvain.be (F.P.); 4 Faculty of Medicine, Imperial College, London SW7 2AZ, UK; giuliamv.iacona@gmail.com; 5 Division of Child Neurology, Department of Pediatrics, Cliniques Universitaires Saint-Luc, Institute of Neuroscience (IoNS), Université Catholique de Louvain, 1200 Brussels, Belgium; roberta.cilio@saintluc.uclouvain.be; 6 Neonatal Sub-Intensive Care Unit and Follow-Up, “Bambino Gesù” Children’s Hospital IRCCS, 00165 Rome, Italy; annabella.braguglia@opbg.net

**Keywords:** perinatal asphyxia, nutritional support, human milk, breastfeeding, donor human milk, feeding tolerance, gastrointestinal outcomes, necrotizing enterocolitis, parenteral nutrition, neonatal intensive care unit, neonatal nutrition

## Abstract

**Background:** Enteral feeding during therapeutic hypothermia (TH) for hypoxic–ischemic encephalopathy (HIE) remains heterogeneous across centres because of concerns regarding feeding intolerance and the risk of necrotizing enterocolitis (NEC). Evidence regarding optimal feeding initiation and advancement remains limited. **Methods:** We performed a secondary analysis of a multicentre retrospective cohort of infants with HIE treated with TH at two tertiary neonatal intensive care units in Italy and Belgium between 2011 and 2024. We included survivors with complete nutritional data, aware that this restriction, adopted because feeding-related endpoints cannot be assessed in non-survivors, may introduce survivor bias. Feeding practices, enteral feeding progression, feeding tolerance, and gastrointestinal outcomes were evaluated. **Results:** Of 302 infants in the original cohort, 276 were included. Enteral feeding during TH was administered to 166 infants (60.1%). Enteral feeding volumes remained low during the first three days of life but progressively increased thereafter. Despite inter-center differences in feeding strategies (initiation and advancement), time to full enteral feeding (median 9 days; IQR 7–12) was comparable overall (Italy: median 9 days, IQR 7–12; Belgium: median 10 days, IQR 7–14; *p* = 0.323). Infants receiving enteral nutrition during TH were observed to achieve full enteral feeding earlier than those not fed during cooling (median 8.0 vs. 12.0 days, *p* < 0.001) and to have a shorter length of hospital stay (median 11.0 vs. 15.0 days, *p* < 0.001). Breastfeeding at discharge was also observed more frequently among infants who received enteral nutrition during TH (79.5% vs. 54.5%, *p* < 0.001). Feeding intolerance occurred in 47 infants (17.0%) and temporary feeding withdrawal in 31 (11.2%). Only three infants (1.1%) developed Bell stage IIA NEC, with no progression to more advanced disease or surgery. NEC was more frequent among infants with Sarnat stage III HIE than among those with stage I–II disease (8.3% vs. 0.4%, *p* = 0.022). **Conclusions:** Enteral feeding during TH was feasible, and gastrointestinal complications were uncommon. These findings support the feasibility of cautious enteral feeding during TH. The concentration of NEC events among infants with severe HIE, although based on only three cases, is hypothesis-generating and suggests that future studies should evaluate feeding strategies according to both neurological severity and physiological stability. Whether earlier initiation or faster advancement is beneficial in clinically stable infants cannot be determined from these observational data and should be addressed in prospective studies.

## 1. Introduction

Hypoxic–ischemic encephalopathy (HIE) is the main neurological consequence of perinatal asphyxia, although multiorgan dysfunction is also common [1]. Therapeutic hypothermia (TH) is currently the standard-of-care treatment for HIE neonates [2,3,4]. Most studies regarding neonates with HIE mainly focused on neuroimaging and neurodevelopmental outcomes, whilst comparatively less attention has been paid to nutritional management during TH and gastrointestinal outcomes [5]. This represents a relevant knowledge gap, given that gastrointestinal involvement is a recognized component of the multiorgan dysfunction associated with perinatal asphyxia.

Historically, enteral feeding during TH has often been withheld because of concerns regarding feeding intolerance and the risk of necrotizing enterocolitis (NEC) [6,7]. These concerns are biologically plausible, as hypoxic–ischaemic injury may impair splanchnic perfusion and intestinal oxygen delivery. Beyond impaired perfusion, restoration of blood flow after the hypoxic insult may itself trigger reperfusion injury, with generation of reactive oxygen species, endothelial dysfunction, and activation of a systemic and local inflammatory response. These mechanisms can further compromise intestinal mucosal integrity and barrier function: they are increasingly recognized as relevant contributors to gastrointestinal injury in asphyxiated neonates [8].

More recent evidence suggests that NEC is uncommon in infants undergoing TH and that enteral feeding during cooling does not increase its occurrence [5,9]. Conversely, enteral feeding during TH may provide potential benefits. Such a protective role might be the consequence of positive effects on structural and functional gut integrity, the enhancement of gut microbiota diversity, and the ability to decrease systemic inflammation [7,10,11]. Despite increasing evidence supporting the safety of enteral feeding during TH, nutritional management remains highly variable across neonatal intensive care units (NICUs) and countries [6,7,10,12]. Indeed, enteral feeding is routinely withheld during TH in some clinical settings [10], whereas in others it is part of standard clinical practice [12].

However, detailed information on enteral feeding practices during TH, including timing of initiation, type and volume of milk, mode of administration, and feeding advancement, remains limited. Moreover, increasing interest in the gut–brain axis provides a biological rationale for investigating whether early nutritional and gastrointestinal management may influence outcomes beyond feeding tolerance itself. However, potential microbiome-mediated or neurodevelopmental effects remain largely hypothetical in this population and require dedicated prospective investigation [13,14].

The aim of this multicentre retrospective study was to describe enteral feeding practices during therapeutic hypothermia in infants with HIE and to evaluate feeding tolerance and gastrointestinal outcomes. We also explored the association between different feeding practices and clinically relevant outcomes, including feeding intolerance, interruption of enteral feeding, and the onset of NEC episodes.

## 2. Methods

### 2.1. Study Design and Outcomes

We conducted a secondary analysis, not prespecified in the protocol of the original study, of a previously established multicentre retrospective cohort evaluating infants with hypoxic–ischemic encephalopathy (HIE) treated with therapeutic hypothermia (TH) at two tertiary neonatal intensive care units: (1) Bambino Gesù Children’s Hospital, Rome, Italy, and (2) Cliniques Universitaires Saint-Luc, Brussels, Belgium [15].

This analysis focused on nutritional management during TH and the early neonatal period.

The primary objective was to describe enteral feeding practices during TH: timing of feeding initiation, route of administration, daily enteral feeding volumes during the first week of life, time to full enteral feeding, and type of milk received. Secondary objectives were to evaluate feeding tolerance, gastrointestinal complications, nutritional outcomes at discharge, and associations between feeding practices and relevant clinical outcomes.

Clinical outcomes included feeding intolerance, interruption of enteral feeding, necrotizing enterocolitis (NEC), hypoglycemic episodes, length of hospital stay, mortality, and breastfeeding status at discharge. Feeding intolerance was defined at both centres based on the presence of gastric residual volume >50% of the previous feed, and/or new-onset abdominal distension, vomiting, or bloody stools. The decision to withhold or temporarily discontinue enteral feeding was based on clinical judgement of the attending team, taking these signs into account together with the infant’s overall clinical condition, rather than on a fixed algorithmic rule.

### 2.2. Setting and Participants

Eligibility criteria and clinical management were identical to those described in the original study. Briefly, infants born at ≥35 weeks’ gestational age with HIE who underwent TH between January 2011 and December 2024 were eligible for inclusion. For the present analysis, only infants who survived to hospital discharge and for whom complete nutritional data were available were included, since discharge-related nutritional outcomes (e.g., time to full enteral feeding, breastfeeding status) could not be assessed in infants who died before discharge; in-hospital mortality was reported separately in the primary cohort study [15]. TH was initiated within 6 h after birth and maintained for 72 h according to local protocols in both centres.

During TH, nutritional management was performed according to local clinical practice. Decisions regarding initiation of enteral feeding, advancement of feeding volumes, use of parenteral nutrition, and selection of milk type (mother’s own milk, donor human milk, or formula) were made by the attending clinical team. In both centers, enteral feeding was generally initiated in infants without haemodynamic instability or need for inotropes, and advanced by approximately 10 mL/kg/day, with feeds withheld or reduced in the presence of gastric residuals >50% of the previous feed volume (if enteral feeding was administered by nasogastric tube), abdominal distension, bilious/bloody residuals, or bloody stools. Routine monitoring of all gastric residuals was abandoned from 2023 in the Italian cohort, and from 2022 in the Belgian cohort.

### 2.3. Variables and Data Collection

For the present secondary analysis, data were extracted from the original multicentre database and supplemented through review of institutional electronic medical records. Variables of interest included demographic and perinatal characteristics (sex, gestational age, birthweight, mode of delivery, Apgar scores, and umbilical cord blood gas parameters), neurological variables (Sarnat stage, electroencephalographic findings, and need for mechanical ventilation), and nutritional variables.

The assessed nutritional variables included administration of enteral nutrition during therapeutic hypothermia, timing and route of feeding initiation, daily enteral feeding volumes during the first week of life, use of parenteral nutrition, time to achievement of full enteral feeding, type of milk received (mother’s own milk, donor human milk, or formula), use of probiotics, and breastfeeding status at discharge.

Clinical outcomes collected during hospitalization included feeding intolerance, temporary withdrawal of enteral feeding, necrotizing enterocolitis (NEC), hypoglycemic episodes (<46 mg/dL), duration of antibiotic therapy, length of hospital stay, and in-hospital mortality.

Data were collected from birth until discharge or death. Selection bias was minimized by including all eligible infants undergoing therapeutic hypothermia during the study period at both participating centres. Information bias was reduced by the use of predefined variables and standardized data extraction procedures. Because of the retrospective nature of the study, some variables were unavailable for all participants; therefore, analyses were performed using available-case data without imputation of missing values.

The study is reported according to the Strengthening the Reporting of Observational Studies in Epidemiology (STROBE) guidelines.

### 2.4. Statistical Analysis

Continuous variables were tested for normality using the Shapiro–Wilk test. Variables with a normal distribution are presented as mean ± standard deviation (SD), whereas non-normally distributed variables are expressed as median and interquartile range (IQR). Categorical variables are presented as numbers and percentages.

Descriptive analyses were performed to characterize feeding practices, nutritional progression, and clinical outcomes during hospitalization. Missing data were not imputed, and percentages were calculated using available observations for each variable.

To account for potential confounding, the association between enteral feeding during TH and time to full enteral feeding, length of hospital stay, and breastfeeding at discharge was further assessed using multivariable linear regression (for the two continuous outcomes) and logistic regression (for breastfeeding at discharge), adjusting for gestational age, birthweight, Sarnat stage, pathological brain MRI, centre, and need for mechanical ventilation. Results were presented as adjusted regression coefficients (β) or odds ratios (aOR), with 95% confidence intervals.

A two-sided *p*-value < 0.05 was considered statistically significant. Statistical analyses were performed using Jamovi (version 2.7.38 for macOS, Sydney, Australia).

### 2.5. Ethical Approval

This retrospective study was conducted using fully anonymized, non-identifiable data and involved no direct patient contact, no additional interventions or therapies beyond routine care. In accordance with the Declaration of Helsinki (6th revision, 2008) and institutional regulations, the study was considered exempt from full ethical review.

The authorization for data publication was given by the Scientific Direction of Bambino Gesù Children’s Hospital (protocol De Rose Domenico Umberto-RAP-2026-0006).

## 3. Results

### 3.1. Study Population

**Figure 1:**
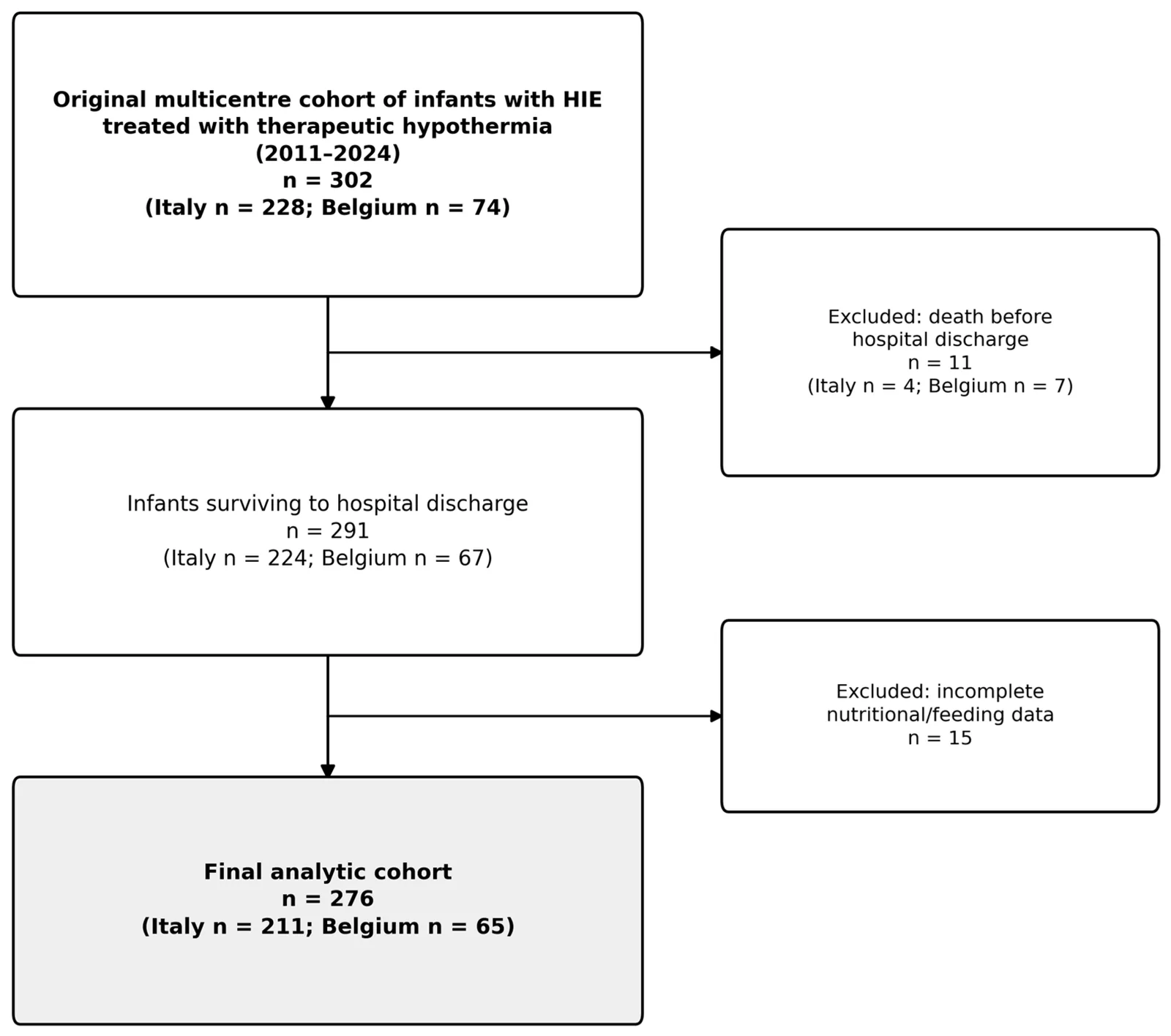
Study flow diagram.

The original multicentre cohort included 302 neonates with HIE treated with TH. For the present secondary analysis, 276 infants were included because nutritional data were not available for all participants enrolled in the primary study (Figure 1). Among survivors (*n* = 291), infants excluded from the present analysis because of incomplete nutritional/feeding data (*n* = 15) did not differ significantly from included infants in Apgar score at 5 min (*p* = 0.379), Sarnat stage (*p* = 0.225), EEG-confirmed seizures (*p* = 0.321), or early exit from TH (*p* = 0.237), supporting the representativeness of the analysed cohort. In particular, no feeding intolerance or NEC episodes were reported in these patients.

Baseline demographic, perinatal, and neurological characteristics of the study population are summarized in Table 1. The majority of infants had moderate hypoxic–ischemic encephalopathy (Sarnat stage II). Mechanical ventilation was required in 108 infants (39.1%). Patent ductus arteriosus was present in 188 infants (68.1%).

### 3.2. Nutritional Management and Feeding Outcomes

Enteral nutrition during hospitalization was characterized by a gradual advancement of enteral feeding volumes throughout the first week of life. It was started during TH in more than half of the infants, whereas parenteral nutrition was administered in most patients during the transition to full enteral feeding.

Full enteral nutrition (150 mL/kg/day) was generally achieved within the first two weeks of life. Feeding intolerance and temporary interruption of enteral nutrition occurred in a minority of infants, while severe gastrointestinal complications were uncommon. Detailed nutritional practices and feeding-related outcomes are summarized in Table 2.

Enteral feeding volumes remained low during the first three days of life in both centres, with a median intake of 0 mL/kg/day on days 1 and 2 (*p* = 0.068 and *p* = 0.738, respectively) and approximately 6 mL/kg/day on day 3 (*p* = 0.532). From day 4 onwards, enteral volumes progressively increased in both centres, without significant differences (*p* = 0.619). By day 7, median enteral intake reached 64.3 mL/kg/day (IQR 38.9–100) in Italian patients and 90.0 mL/kg/day (IQR 52.4–116) in Belgian patients (*p* = 0.003).

**Figure 2:**
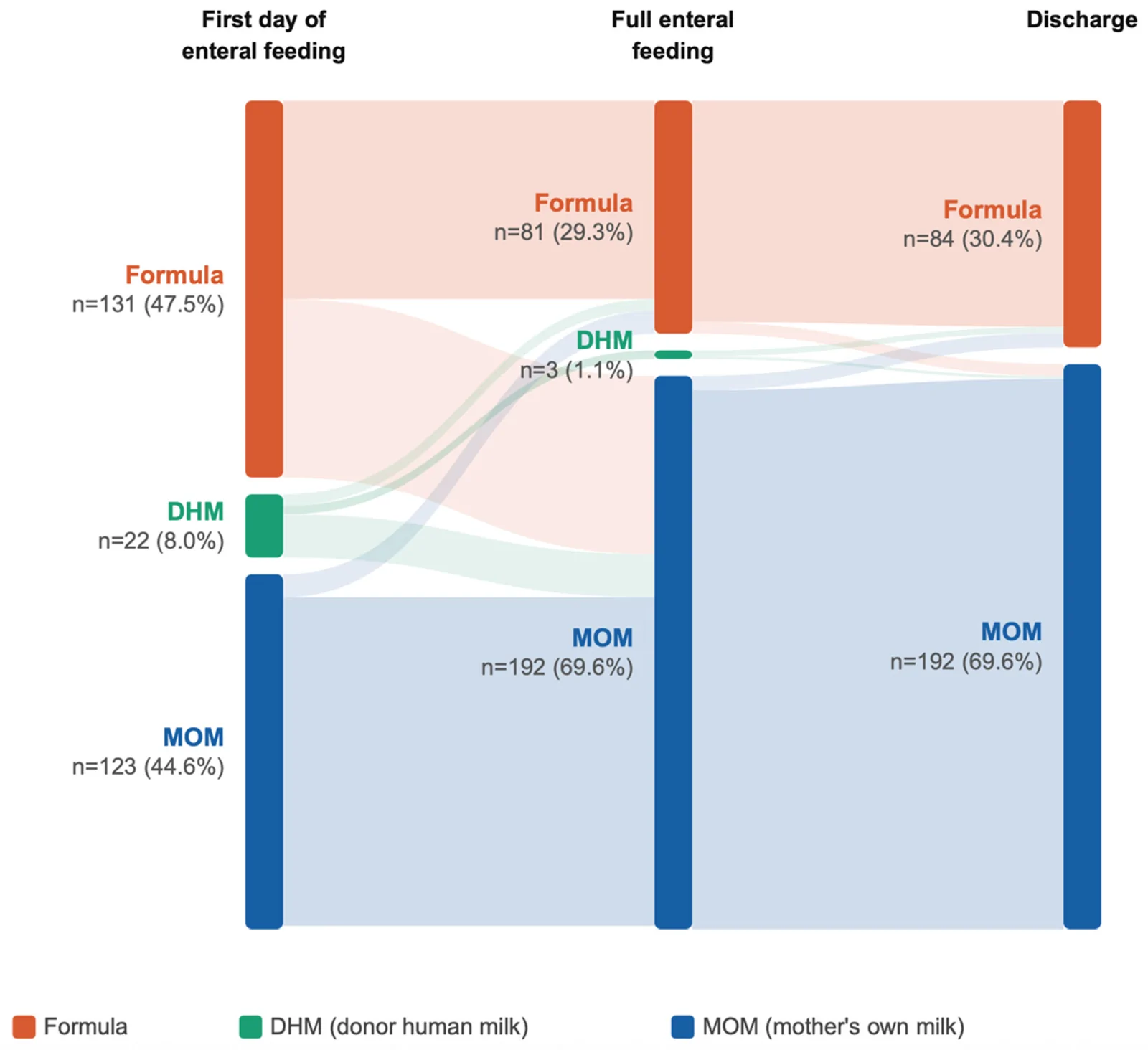
Sankey diagram illustrating the transitions in milk type across three feeding stages in the discharged patients (*n* = 276). Each of the three vertical bars corresponds to one time point (first day of enteral feeding, full enteral feeding, and discharge); the height of each coloured segment within a bar is proportional to the number of infants receiving formula, donor human milk (DHM), or mother’s own milk (MOM) at that time point. The coloured flows (ribbons) connecting the bars trace how individual infants moved between milk types from one stage to the next, so that the width of a flow reflects the number of infants following that particular trajectory. DHM was not available as a feeding option at discharge; the two infants receiving DHM at full enteral feeding were transitioned to formula prior to discharge.

A progressive shift towards human milk feeding was observed during hospitalization. Although formula was commonly used at the beginning of enteral nutrition, mother’s own milk became increasingly prevalent during the NICU stay and represented the predominant feeding modality both at full enteral feeding and at discharge (Figure 2).

Only three infants (1.1%) developed Bell stage IIA NEC episodes during hospitalization, diagnosed by the attending neonatologist based on clinical presentation and abdominal radiographs. All cases presented with bloody stools. The radiological evidence of pneumatosis intestinalis was confirmed by an independent radiologist. In all three cases, there was no progression to more advanced disease. Given the very low number of NEC cases (*n* = 3), only exploratory univariate analyses were performed, and effect estimates were not considered robust enough for multivariable modelling. Among the three infants who developed NEC, two had feeding intolerance, and two required temporary withdrawal of enteral feeding.

NEC occurred in 2 of 24 infants (8.3%) with Sarnat stage III encephalopathy and in 1 of 252 infants (0.4%) with Sarnat stage I–II encephalopathy (*p* = 0.022).

No association was found between NEC occurrence and enteral feeding during therapeutic hypothermia (*p* = 0.817), type of milk received at full enteral feeding (*p* = 0.349), mechanical ventilation (*p* = 0.343), sex (*p* = 0.150), mode of delivery (*p* = 0.447), or antibiotic prophylaxis at birth (*p* = 0.567). Although NEC occurred only in infants receiving proton pump inhibitors (3/102, 2.9%) rather than among those not exposed (0/174) (*p* = 0.049), the very small number of NEC cases precludes any firm conclusions regarding a causal association. None of the 22 infants receiving probiotics during NICU admission developed NEC, compared with 3 of 254 infants (1.2%) who did not receive probiotics (*p* = 1.000).

Infants receiving enteral nutrition during TH were observed to achieve full enteral feeding earlier than those not fed during cooling (median [IQR], 8.0 [6.75–10.3] vs. 12.0 [9.0–19.0] days, *p* < 0.001) and to have a shorter length of hospital stay (median [IQR], 11.0 [9.0–14.0] vs. 15.0 [12.0–24.8] days, *p* < 0.001).

After adjustment for gestational age, birthweight, Sarnat stage, pathological brain MRI, centre, and need for mechanical ventilation, enteral feeding during TH remained independently associated with earlier achievement of full enteral feeding (adjusted β = −3.7 days, 95% CI −5.8 to −1.5, *p* < 0.001; R^2^ = 0.221), indicating that this association persisted even after accounting for markers of illness severity and centre (Table 3). Mechanical ventilation (β = 2.8 days, *p* = 0.016) and pathological brain MRI (β = 2.9 days, *p* = 0.007) were independently associated with a longer time to full feeds, whereas centre was not significantly associated after adjustment (*p* = 0.142).

After adjustment for the same 6 variables, enteral feeding during TH also remained independently associated with a shorter length of hospital stay (adjusted β = −6.0 days, 95% CI −10.1 to −1.9, *p* = 0.004; R^2^ = 0.166). Pathological brain MRI (β = 5.9 days, *p* = 0.004) and mechanical ventilation (β = 5.1 days, *p* = 0.020) were independently associated with a longer hospital stay, whereas centre was not significantly associated after adjustment (*p* = 0.465).

Furthermore, breastfeeding at discharge was observed more frequently among infants who received enteral nutrition during therapeutic hypothermia than among those who did not (132/166, 79.5% vs. 60/110, 54.5%; *p* < 0.001).

After adjustment for gestational age, birthweight, Sarnat stage, pathological brain MRI, centre, and need for mechanical ventilation, enteral feeding during TH remained independently associated with breastfeeding at discharge (adjusted OR 2.52, 95% CI 1.36–4.67, *p* = 0.003; Nagelkerke R^2^ = 0.099). None of the other covariates were independently associated with breastfeeding at discharge in the adjusted model.

## 4. Discussion

In this multicentre cohort of neonates undergoing TH for HIE, gastrointestinal complications were infrequent, and NEC was rare. More than half of the infants received enteral feeds during TH, full enteral feeding was generally achieved within the first two weeks of life, and approximately 70% received mother’s own milk at discharge. Although enteral feeding during TH was used with similar frequency and was introduced cautiously in both participating centres, nutritional practices differed between centres. Belgian neonates were more frequently fed during TH, received larger enteral volumes during the first week of life, and were exposed less frequently to proton pump inhibitors (PPIs). Italian neonates had fewer reported hypoglycemic episodes, and a small proportion received probiotics. At feeding initiation, 46% of neonates in Italy were fed directly orally, whereas all neonates in Belgium were fed via a nasogastric tube. Median enteral volumes were negligible during the first two days of life and remained low on day 3, consistent with a minimal enteral feeding approach during TH. After rewarming, feeding volumes increased progressively in both centres, with a numerically faster advancement in the Belgian centre. Time to full enteral feeding was similar between centres, despite this faster early advancement in Belgium. Nevertheless, gastrointestinal outcomes were similar between centres. Rates of feeding intolerance, temporary interruption of enteral feeding, necrotizing enterocolitis, and breastfeeding at discharge did not differ significantly between centres. Although the retrospective design and the long study period prevent attribution of any difference to centre-specific policies, these findings suggest that different nutritional strategies may be associated with comparable clinical outcomes and raise the possibility that a more proactive approach to enteral feeding may be feasible in selected clinically stable infants.

In line with this, Costa et al. reported that minimal enteral feeding during TH was independently associated with earlier achievement of full enteral feeding and shorter duration of parenteral nutrition, suggesting that even small enteral volumes during cooling may facilitate subsequent nutritional progression [16].

Historically, enteral feeding during TH has often been conservative because of concerns regarding impaired perfusion, feeding intolerance, and NEC. Consequently, many neonatal units delayed or completely withheld enteral nutrition throughout the cooling period. Early therapeutic hypothermia trials were generally conservative regarding enteral nutrition; in the TOBY trial, enteral feeding was withheld during cooling, reflecting concerns that hypoxic–ischaemic injury and reduced splanchnic perfusion could increase the risk of gastrointestinal complications [2]. However, growing evidence has challenged this paradigm. UK practice subsequently shifted towards cautious enteral feeding during TH: the TOBY Register handbook recommended the cautious introduction of enteral feeds after the first 24 h of life, and a national survey found that 59% of neonatal units were already providing enteral feeds during cooling or rewarming, albeit with substantial variability in timing, volumes, and feeding advancement [6].

This variability was subsequently highlighted by Chandrasekaran et al., who emphasized the lack of randomized evidence supporting routine withholding of enteral feeds during TH and the need for evidence-based nutritional strategies in this population [17]. Large observational studies and recent systematic reviews consistently suggest that enteral feeding during TH is not associated with an increased risk of severe gastrointestinal complications and may facilitate earlier nutritional recovery. Our findings are consistent with this evolving evidence. In a large UK population-based study including more than 6000 cooled infants, severe NEC was extremely rare, occurring in only seven infants, while enteral feeding during TH was associated with favourable secondary outcomes, including earlier establishment of breastfeeding and shorter duration of parenteral nutrition and hospital stay [7]. More recently, a systematic review and meta-analysis including 3693 infants found an overall incidence of stage II–III NEC of only 0.6%, with no evidence of an increased risk among enterally fed infants [5].

Importantly, our study extends previous observations by comparing two centres adopting different nutritional strategies. Differences in feeding practices, including earlier initiation and faster advancement of enteral nutrition, were not accompanied by worse gastrointestinal outcomes. Despite a significantly earlier introduction of enteral feeds in the Belgian centre, time to full enteral feeding remained similar, and rates of feeding intolerance and NEC were comparable.

In our cohort, feeding intolerance occurred in 17.0% of infants. This rate was lower than the 28.8% reported by Hu et al. in the first randomized controlled trial specifically evaluating feeding intolerance in infants with HIE undergoing TH [18]. However, differences in the reported incidence of feeding intolerance across studies should be interpreted with caution, as they may partly reflect differences in its definition and in the clinical criteria used to identify feeding intolerance. Hu et al. found no increase in feeding intolerance with early enteral nutrition (during TH/rewarming) compared with delayed enteral nutrition (after TH) (26.2% vs. 31.6%, *p* = 0.595), suggesting that feeding intolerance, although relatively common in this population, may not necessarily be driven by early enteral feeding itself [18].

We observed a low incidence of NEC despite more than half of the cohort receiving enteral feeding during TH. Only three infants (1.1%) developed Bell stage IIA NEC, and none progressed to more advanced disease or surgery.

Although our retrospective design precludes causal inference, the low incidence of NEC observed in this cohort is reassuring. However, given the very small number of events, our data do not allow conclusions regarding the comparative safety of specific feeding strategies and should not be interpreted as demonstrating that a less restrictive feeding strategy is safe in all infants. The very low number of events precludes any analysis of NEC risk factors and primarily highlights the rarity of this complication in our cohort. The observational design does not allow us to determine whether one feeding strategy is preferable to another or to define the optimal timing, volume, or rate of feeding advancement.

Nevertheless, the clustering of NEC among infants with severe HIE raises an important hypothesis regarding patient selection for feeding during TH. Two of the 24 infants with Sarnat stage III HIE developed NEC, compared with only one of 252 infants with stage I–II disease. Because only three NEC events occurred, this observation should not be interpreted as evidence that Sarnat stage independently predicts NEC or as justification for withholding enteral feeds in infants with severe HIE. Rather, neurological severity may partly reflect the extent of the systemic hypoxic–ischaemic insult, which may plausibly influence gastrointestinal tolerance. From a clinical perspective, these findings suggest that feeding strategies could be tailored according to clinical severity. In clinically stable infants without evidence of haemodynamic compromise, earlier initiation and more rapid advancement of enteral feeding might be feasible, whereas greater caution may be warranted in infants with severe HIE and haemodynamic compromise. To our knowledge, this concept is insufficiently addressed in the current literature: nutritional management during therapeutic hypothermia should probably not be considered a binary decision of “feeding” versus “not feeding”. The severity of hypoxic–ischaemic injury, haemodynamic stability, vasoactive support, evidence of impaired systemic perfusion, and gastrointestinal perfusion are all likely to influence the safety of enteral nutrition. Our observation that NEC occurred predominantly among infants with severe HIE, although based on very few events, raises the possibility that a risk-adapted approach may be more appropriate than a uniform feeding policy for all infants undergoing TH. Importantly, our data cannot define the clinical thresholds that should guide such decisions. Prospective studies should therefore examine neurological severity together with dynamic markers of physiological stability, including vasoactive support, systemic perfusion and, where available, indices of gastrointestinal perfusion.

Differences between centres also illustrate a broader implementation-science phenomenon [19]. Even when the underlying evidence base is similar, local unit culture, clinician equipoise, and historical practice patterns can lead to persistently different feeding strategies. This suggests that translating reassuring observational and trial evidence into more uniform bedside practice may require dedicated implementation strategies (e.g., shared protocols, audit-and-feedback), rather than publication of evidence alone. Differences between centres should also be interpreted in the context of the long study period. Nutritional management evolved between 2011 and 2024, and changes did not necessarily occur simultaneously in the two institutions. Earlier initiation of feeding and differences in feeding advancement, use of PPIs or probiotics, availability of donor human milk, and policies regarding gastric residual monitoring are examples of practices that changed over time. Because temporal trends were not formally evaluated in the present secondary analysis, centre and calendar period cannot be fully disentangled.

PPI use was associated with a higher frequency of feeding intolerance in unadjusted analyses; however, PPIs were often started after blood-stained gastric residuals were observed, and this association is likely affected by confounding by indication. Given the retrospective design and the very small number of NEC events, our data cannot establish, and are not intended to establish, whether PPI exposure causally contributes to gastrointestinal morbidity in these newborns. In neonatal populations, acid suppression has been associated with intestinal dysbiosis and an increased risk of NEC and infections [20,21]. We regard the association observed in our cohort as hypothesis-generating only, and it should prompt prospective evaluation of PPI use in this population rather than an immediate change in clinical practice.

Concerning probiotics, although they were used only in the Italian centre and none of the treated infants developed NEC, the very small number of NEC events prevents any conclusions regarding a protective effect.

An additional finding concerned the type of enteral nutrition. Although formula was frequently used when enteral feeding was initiated, mother’s own milk progressively became the predominant source of nutrition during hospitalization, highlighting both its feasibility and its central role in the nutritional management of infants with HIE. The proportion of infants receiving mother’s own milk at discharge was similar between centres and higher than that reported in some previous cohorts [22]. The lower availability of mother’s own milk during the early phase may reflect several challenges previously described in this population, including maternal–infant separation, delayed lactogenesis following acute perinatal stress, and difficulties in initiating and maintaining milk expression [23]. In particular, the association between feeding during TH and breastfeeding at discharge should be interpreted cautiously, as it is unlikely to be solely explained by feeding during cooling itself. Unmeasured factors such as the intensity of lactation support received, overall illness severity, and duration of maternal–infant separation are plausible confounders that could not be fully accounted for in this retrospective analysis. All these findings highlight the importance of providing early lactation support to mothers of infants undergoing TH.

Beyond its nutritional value, human milk may provide important biological advantages in this particularly vulnerable population. Its anti-inflammatory, immunomodulatory, and trophic properties may help preserve intestinal barrier integrity, improve intestinal perfusion and epithelial repair, promote the establishment of a healthy gut microbiome, and reduce intestinal inflammation [24,25,26]. Experimental and clinical evidence suggests that the gut microbiome and its metabolites may modulate neuroinflammatory pathways through the gut–brain axis [14,27,28,29]. This provides a biological rationale for investigating whether early nutritional strategies and human milk exposure might influence neurological recovery after hypoxic–ischaemic injury. However, microbiome composition, neuroinflammatory biomarkers, and neurodevelopmental outcomes were not evaluated in the present study; therefore, any potential neurological effect of feeding or human milk remains hypothetical and should be addressed in dedicated prospective studies.

From a clinical perspective, human milk should remain the preferred enteral feed whenever available, consistent with current neonatal nutritional practice. In our cohort, donor human milk (DHM) was used exclusively in the Italian centre, whereas it was reserved for preterm infants in the Belgian centre. Although our study did not provide evidence regarding the potential benefits of DHM in this population, its use may represent a reasonable alternative to formula milk when mother’s own milk is unavailable, given the potential biological advantages of human milk.

The strengths of this study include its multicentre design, the relatively large cohort of infants undergoing TH, and the detailed longitudinal characterization of enteral feeding practices during the first week of life, which allowed comparison of different real-world nutritional approaches between centres. Several limitations should also be acknowledged. First, the retrospective design may have resulted in incomplete data collection and unmeasured confounding. Infants who died before discharge, predominantly the most severely affected, were excluded from the present nutritional analysis. Mortality was higher in the Belgian cohort; however, all deaths in this centre occurred within the first days of life following redirection of care, agreed with the families because of evidence of severe brain damage, rather than because of gastrointestinal or nutrition-related complications. We therefore consider it unlikely that this exclusion biased the feeding-outcome associations reported here toward more favourable results in the fed group, although this cannot be entirely excluded. Second, nutritional management was not standardized and reflected local clinical practice. The study spans a long 14-year period (2011–2024), during which feeding strategies and several components of supportive nutritional care evolved. These included donor human milk availability, probiotic and PPI use, feeding advancement practices, and policies regarding routine assessment of gastric residuals. Importantly, such changes may not have occurred at the same time in the two centres. Therefore, some of the differences attributed to centre may instead reflect temporal changes in practice or centre-by-era effects. We did not formally analyse temporal trends or adjust the main models for calendar period, and temporal confounding must therefore be considered when interpreting both inter-centre comparisons and feeding–outcome associations. Although this introduces potential confounding, the long observation period also allowed us to describe and compare different real-world feeding approaches over time. Third, the very low number of NEC cases limited statistical power and precluded formal assessment of factors associated with NEC or multivariable modelling. Detailed measures of haemodynamic instability and disease severity during the acute phase (e.g., inotropic/vasopressor support, systemic perfusion indices) were not systematically collected in the original database and could therefore not be included as covariates; residual confounding by these unmeasured variables cannot be excluded and should be considered when interpreting the observed associations between feeding during TH and clinical outcomes. Finally, the decision to initiate enteral feeding during TH was not randomized but reflected clinical judgement based on the infant’s perceived stability; residual confounding by indication is therefore likely the most important limitation of this study, and the associations reported between feeding during TH and favourable outcomes may partly reflect underlying differences in illness severity rather than a causal effect of feeding itself.

## 5. Conclusions

Enteral feeding during TH was feasible in this multicentre cohort, while severe gastrointestinal complications were uncommon. Enteral nutrition during TH was associated with earlier achievement of full enteral feeding, shorter hospital stay, and more frequent breastfeeding at discharge, compared with infants not fed during cooling. Given the retrospective design and the likelihood that infants selected for feeding during TH were clinically more stable, these associations should not be interpreted as causal. Human milk should remain the first choice for enteral feeding, whenever available, and efforts to support lactation and maternal milk expression should be encouraged, including when mother-infant separation occurs during TH. Together with previous evidence, our findings support the feasibility of cautious enteral feeding during TH in infants with HIE. Clinical decision-making should therefore move beyond the binary question of feeding versus fasting during therapeutic hypothermia. The timing of initiation and rate of advancement may need to be individualized according to physiological stability and the severity of the systemic hypoxic–ischaemic insult. The clustering of NEC among infants with severe HIE in our cohort is hypothesis-generating but, given the very small number of events, does not establish Sarnat stage as a criterion for withholding or delaying enteral feeding.

Future randomized studies should determine whether earlier or faster feeding advancement improves outcomes, moving beyond simply comparing feeding versus fasting during therapeutic hypothermia. Such studies should also clarify which infants are most likely to benefit from earlier nutritional advancement, which clinical parameters should guide feeding progression, and whether the type of milk influences gastrointestinal and neurological outcomes in infants undergoing therapeutic hypothermia.

## Figures and Tables

**Table 1:** Clinical characteristics of newborns participating in the study.

Characteristics	All Included Patients (*n* = 276)	Italian Patients (*n* = 211)	Belgian Patients (*n* = 65)	*p*-Value
Male sex, *n* (%)	164 (59.4%)	122 (57.8%)	42 (64.6%)	0.329
Gestational age, weeks, median (IQR)	39 (38–40)	39 (38–40)	39 (37–40)	0.155
Birthweight, g, mean ± SD	3207 ± 556	3205 ± 521	3213 ± 660	0.924
Cesarean delivery, *n* (%)	124 (44.9%)	88 (41.7%)	36 (55.4%)	0.053
Apgar score at 5 min, median (IQR)	6 (4–7)	6 (5–7)	4 (3–5)	**<0.001**
Umbilical cord pH, median (IQR)	7.02 (6.90–7.14)	7.03 (6.91–7.14)	6.98 (6.90–7.14)	0.226
Umbilical cord Base Excess, mmol/L, median (IQR)	−15.9 (−19.4–−13.2)	−16.0 (−19.8–−13.5)	−14.8 (−17.0–−12.4)	0.046
Umbilical cord Lactate, mmol/L, median (IQR)	11.0 (7.95–14.4)	10.9 (8.47–14.8)	12.1 (5.47–14.1)	0.333
Sarnat stage I, *n* (%)	10 (3.6%)	0	10 (15.4%)	**<0.001**
Sarnat stage II, *n* (%)	242 (87.7%)	191 (90.5%)	51 (78.5%)	
Sarnat stage III, *n* (%)	24 (8.7%)	20 (9.5%)	4 (6.1%)	
EEG-confirmed seizures, *n* (%)	70 (26.0%)	54 (25.6%)	16 (24.6%)	0.874
Pathological brain MRI, *n* (%)	108 (39.3%)	81 (38.4%)	27 (41.5%)	0.649
Early exit from TH, *n* (%)	6 (2.2%)	6 (2.8%)	0	0.169
Length of stay, days, median (IQR)	12 (10–17)	12 (10–17)	13 (8–16)	0.554

*Missing data: Umbilical cord pH, n = 9; Umbilical cord Base excess, n = 28; Umbilical cord Lactate, n = 60.*

**Table 2:** Nutritional management and feeding outcomes during NICU stay.

Characteristics	All Included Patients (*n* = 276)	Italian Patients (*n* = 211)	Belgian Patients (*n* = 65)	*p*-Value
Enteral nutrition during therapeutic hypothermia, *n* (%)	166 (60.1%)	118 (55.9%)	48 (73.8%)	**0.002**
Nasogastric tube feeding at initiation, *n* (%)	179 (64.9%)	114 (54.0%)	65 (100%)	**<0.001**
Timing of enteral feeding initiation, days, median (IQR)	3 (2–4)	3 (2–4)	2 (1–3)	**<0.001**
Days to full enteral feeding, median (IQR)	9 (7–12)	9 (7–12)	10 (7–14)	0.323
Parenteral nutrition, *n* (%)	261 (94.9%)	196 (92.9%)	65 (100%)	**0.027**
Feeding intolerance, *n* (%)	47 (17.0%)	33 (15.6%)	14 (21.5%)	0.269
Withdrawal of enteral feeding, *n* (%)	31 (11.2%)	22 (10.4%)	9 (13.8%)	0.445
NEC episodes, *n* (%)	3 (1.1%)	2 (0.9%)	1 (1.5%)	0.555
Hypoglycemia, *n* (%)	35 (12.7%)	13 (6.2%)	22 (33.8%)	**<0.001**
Probiotics administration, *n* (%)	22 (8.0%)	22 (10.4%)	0	**0.004**
Proton pump inhibitor (PPI) use, *n* (%)	102 (37.0%)	93 (44.1%)	9 (13.8%)	**<0.001**
Breastfeeding at discharge, *n* (%)	192 (69.6%)	146 (69.2%)	46 (70.7%)	0.809

**Table 3:** Multivariable analysis of the association between enteral feeding during therapeutic hypothermia (TH) and major clinical outcomes.

Outcome	Unadjusted Comparison (Fed vs. Not Fed During TH)	Adjusted Estimate * (95% CI)	Adjusted *p*	Model R^2^
Time to full enteral feeding (days)	Median 8.0 vs. 12.0 (***p* < 0.001**)	β = −3.7 (−5.8 to −1.5)	**<0.001**	0.221
Length of hospital stay (days)	Median 11.0 vs. 15.0 (***p* < 0.001**)	β = −6.0 (−10.1 to −1.9)	**0.004**	0.166
Breastfeeding at discharge, *n* (%)	132/166 (79.5%) vs. 60/110 (54.5%) (***p* < 0.001**)	aOR = 2.52 (1.36–4.67)	**0.003**	0.099 ^a^

* Adjusted for gestational age, birthweight, Sarnat stage, pathological brain MRI, centre, and need for mechanical ventilation. Linear regression models are reported as adjusted regression coefficients (β, in days); the breastfeeding model is reported as adjusted odds ratio (aOR). ^a^ Nagelkerke R^2^ (logistic regression); R^2^ for the linear regression models.

## Data Availability

All data relevant to the study are included in the article. Other details are available upon reasonable request.
